# Case Report: Bilateral perineuroma of the orbit presenting as vertical binocular diplopia

**DOI:** 10.3389/fopht.2026.1777572

**Published:** 2026-03-13

**Authors:** Amanda K. Hertel, Robert Boyle, Jason A. Sokol

**Affiliations:** Department of Ophthalmology, University of Kansas School of Medicine, Kansas City, KS, United States

**Keywords:** diplopia, orbit, orbital tumor, perineuroma, peripheral nerve sheath tumor

## Abstract

In this case report, we describe a 65-year-old woman who presented to the ophthalmology clinic with vertical binocular diplopia for approximately 2 years. Visual exam was notable for bilateral proptosis and ophthalmoplegia. A computed tomography (CT) of the head revealed bilateral enhancing orbital masses. A biopsy was performed, and the result was positive for epithelial membrane antigen (EMA) and CD34, confirming a diagnosis of perineuroma. To our knowledge, this is the first reported case of bilateral perineuroma of the orbit, a rare location for an already uncommon benign tumor.

## 1 Introduction

Perineuroma is an uncommon type of peripheral nerve sheath tumor (PNST) made of perineurial cells, typically found in adults. Two main types exist: intraneural (peripheral nerve boundaries) and extraneural (soft tissues and skin) (1). Most commonly, extraneural perineuromas occur in subcutaneous tissues of the trunk or limbs. Much less commonly, they may occur in the head and neck, retroperitoneum, brain, kidneys, or intestines (1). Perineuroma of the orbit has been documented but is uncommon (1–4).

In this case report, we review a rare case of perineuroma presenting as bilateral orbit masses.

## 2 Case presentation

A 65-year-old woman with a medical history of breast cancer (ER+, PR+, HER2−; poorly differentiated ductal carcinoma of the right breast, status post lumpectomy, treated with hormonal letrozole and adjuvant radiation therapy; without metastasis) and chronic kidney disease (CKD) stage 3a, presented to the neuro-ophthalmology clinic in July 2022 with a chief complaint of vertical binocular diplopia. This diplopia began after a fall in October 2020, in which she injured several upper extremity bones, but none in the face or orbits. She then presented to the emergency department (ED) and was referred to orthopedic surgery, but had not received any head or facial imaging. She had additional symptoms, including dysphagia, difficulty walking, and muscle spasms along her right lower extremity during her neuro-ophthalmology evaluation.

**Figure 1:**
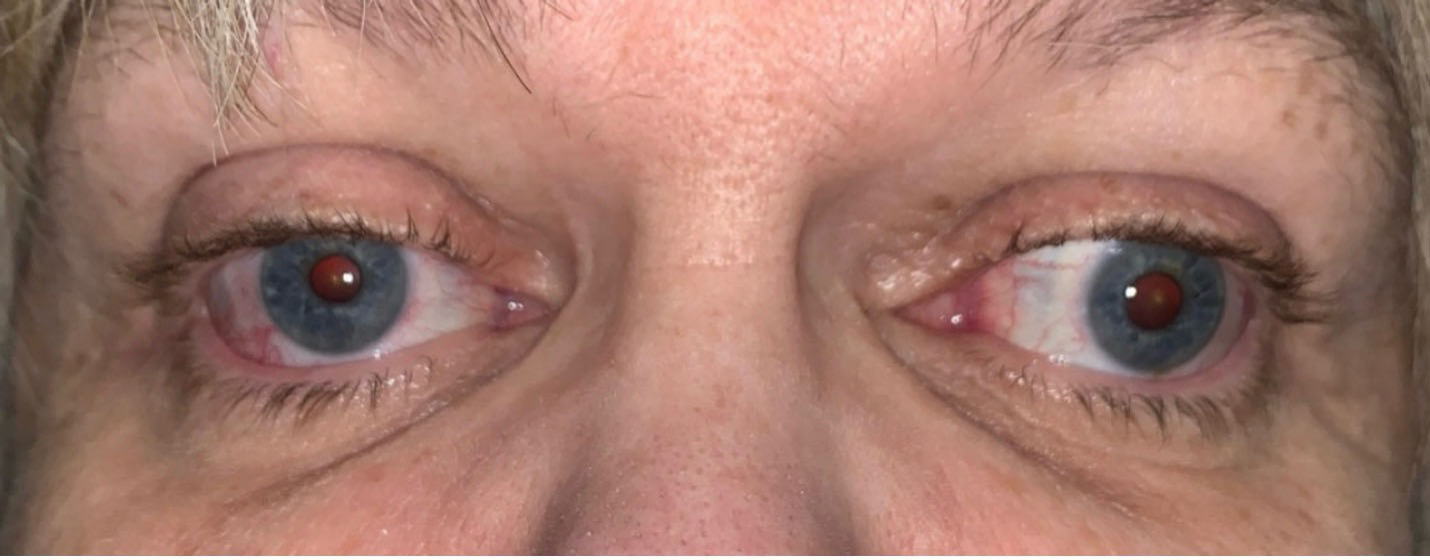
External image showing proptosis, ptosis, and ophthalmoplegia due to the bilateral perineuromas.

On initial clinic presentation, she exhibited a chin-up posture. Her visual acuity (VA) was 20/25 −1 in the right eye (OD) and 20/25 −3 in the left eye (OS). Tonometry using an iCare revealed intraocular pressures (IOP) of 12 mmHg OD and 10 mmHg OS. Pupils were equal, round, and briskly reactive to light, bilaterally measuring 4 mm. There was no afferent pupillary defect. Color testing using Hardy–Rand–Rittler plates showed potential trace red–green color loss OD with normal color vision OS. Results of the cranial nerve (CN) exam were normal for CN V, VII, VIII, IX, X, and XII. The slit lamp exam revealed a 2- to 3-mm ptosis in both eyes (OU), modest nuclear sclerotic (NS) cataracts OU, and vitreous syneresis OU. The fundus exam showed no abnormalities. The margin reflex distance 1 (MRD1) was 3 mm OU, the palpebral fissure was 7 mm OU, and levator function was not assessed. Additionally, there was decreased supraduction, abduction, and adduction of both globes that did not improve with oculocephalic head movements, suspicious for external ophthalmoplegia (**Figure 1**). Infraduction was normal OU. On prism and cover testing, the patient demonstrated 10 prism diopters (PD) exotropia and 8–10 PD right hypertropia in primary gaze. In the clinic, the patient exhibited suppression on the Worth 4 Dot test and declined prism glasses. Humphrey visual fields showed an enlarged blind spot OD and a superior nasal defect OS but had poor reliability (**Supplementary Figure 1**).

A contrast-enhanced CT of the orbits was ordered and performed, which showed bilateral enhancing orbital masses and mild proptosis. There were no bone changes. The lesions were extraconal and oriented along the expected course of the supraorbital and supratrochlear nerves, favoring a neurogenic origin prior to biopsy. A magnetic resonance imaging (MRI) of the head with and without contrast was performed in 2017 due to right-sided weakness and balance issues. This prior imaging suggested that the masses had been there since then, with little change. Differential considerations at this time included lymphoma, metastatic disease, and idiopathic or granulomatous orbital inflammatory disease. Laboratory tests were ordered, including thyroid-stimulating hormone (TSH), thyroid-stimulating immunoglobulin (TSI), thyroxine (T4), angiotensin-converting enzyme (ACE), interleukin-2 (IL-2) receptor, erythrocyte sedimentation rate (ESR), C-reactive protein (CRP), and immunoglobulin G (IgG) antibodies with subclasses (IgG1, IgG2, IgG3, and IgG4). Laboratory tests revealed an elevated IL-2 receptor [1,067.3, normal (175.3, 858.2)], low IgG2 [140, normal (171, 632)], low IgG3 [18.1, normal (18.4, 106.0)], and IgG4 [0.9, normal (2.4, 121.0)].

The patient was then referred to an oculoplastic surgeon in 2022 for biopsy. A right lateral orbitotomy with bone window and medial rectus biopsy were performed. Histologic analysis showed proliferation of bland, elongated spindle cells in various growth patterns (storiform, lamellar, and fascicular) admixed in collagenous to focally myxoid stroma. Immunostains were positive for epithelial membrane antigen (EMA) and CD34. The tissue also stained negative for S100, SOX10, signal transducer and activator of transcription 6 (STAT6), somatostatin receptor 2 (SSTR2), pan keratin, and smooth muscle actin (SMA). The cells did show a proliferative rate with Ki-67 immunostain. The Cleveland clinic performed a sarcoma fusion next-generation sequencing (NGS) panel, which was negative. Notably, GLUT-1 and claudin 1 immunostains were negative. At this point, the orbital masses were determined to be perineuromas based on their morphological features and positivity for EMA and CD34.

**Figure 2:**
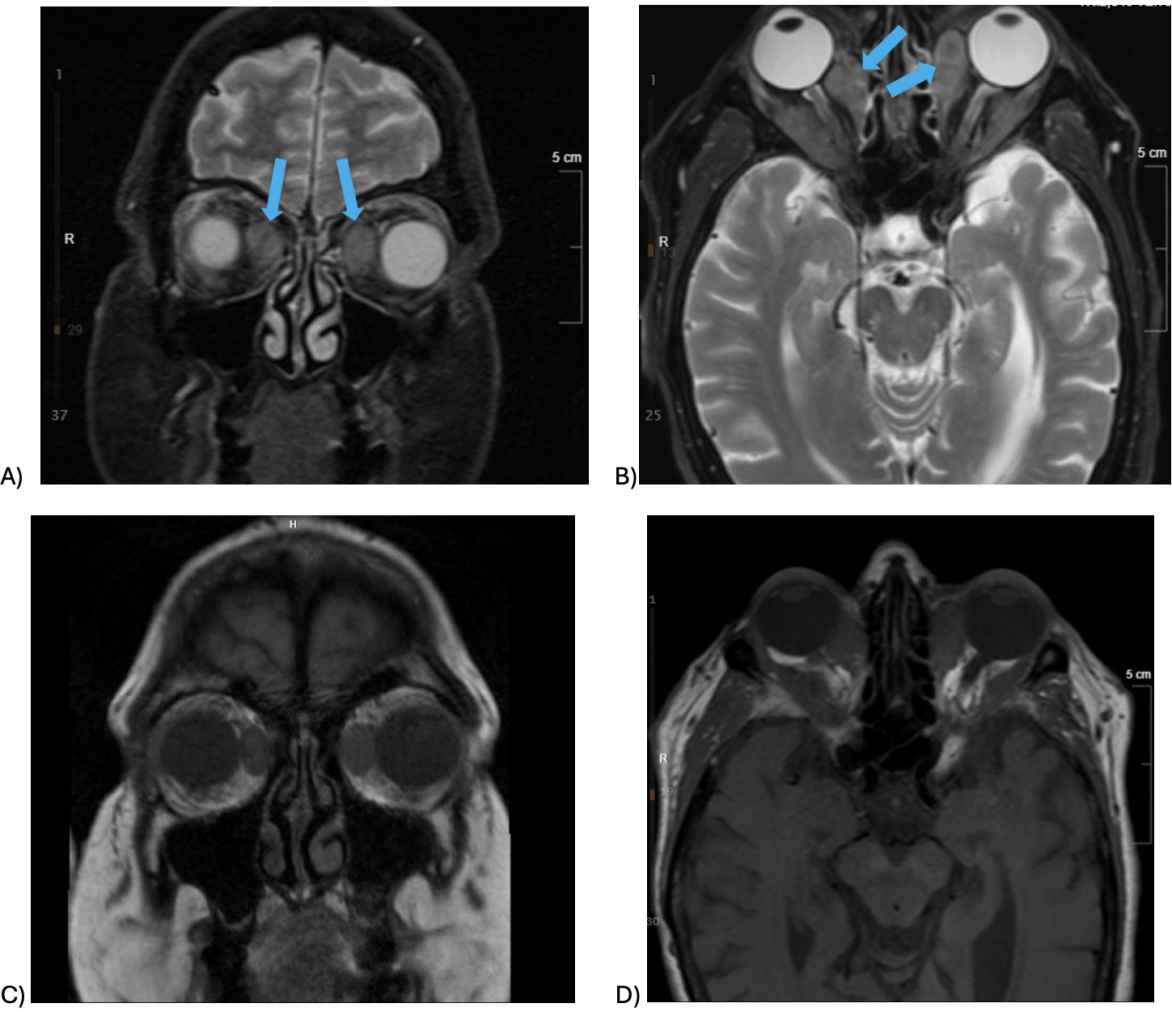
**(A–D)** MRI orbit without contrast performed in December 2022 showing bilateral perineuromas. **(A)** T2-weighted coronal view, **(B)** T2-weighted axial view, **(C)** T1-weighted coronal view, and **(D)** T1-weighted axial view.

A referral was placed for medical oncology. Additional work-up was ordered and completed in 2022. An MRI of the orbits showed symmetric bilateral orbital masses and mild proptosis, similar to previous CT findings (**Figures 2A–D**). The bilateral lesions were described as lobulated homogeneously enhancing conal–extraconal orbital mass lesions, with poor delineation from the extraocular musculature. There was a relatively symmetric T2 hypointense enhancement. The lesions involved medial rectus muscles bilaterally as well as insinuating circumferentially along the deep coronal margins of the orbits, with distal extension into the bilateral orbital roofs and the temporal and inferior extraconal orbit. The lesions appear to compress the orbital fat and crowd the optic nerves at the orbital apex. Brain MRI showed no acute findings or masses. Because of the unique presentation of bilateral perineuromas, a full-body MRI was performed in case there were additional perineuromas. On full-body MRI, there was a probable small vertebral hemangioma at T1, but no other soft tissue masses or lymphadenopathy. Genetic testing showed no variants. Genes tested included the following: ATM, BARD1, BRCA1, BRCA2, BRIP1, CDC73, CDH1, CHEK2, DICER1, LZTR1, MLH1, MRE11A, MSH2, MSH6, NBM, NF1, NF2, PALB2, PMS2, PRKAR1A, PTEN, RAD51D, RECQL, SDHB, SDHD, SMARCA4, SMARCB1, STK11, TP53, FAM175A, RINT1, and EPCAM.

In late 2022, she consulted radiation oncology, but given the stability of the lesions on imaging and the degree of symptoms, they elected for observation at that time. This was also largely due to the risk of vision loss with any surgical management. Oral steroids were administered, which only helped with muscle weakness temporarily at the higher 40-mg dose, but also led to side effects, including insomnia.

In December 2024, the patient developed worsening of clinical symptoms despite stable imaging. At this time, the exam revealed a VA of 20/20 OU and an IOP of 12 OD and 9 OS. The ophthalmoplegia had worsened now with –2 infraduction deficits in addition to stable but reduced supraduction, abduction, and adduction OU. These symptoms include discomfort in her right eye, pain behind both eyes, and decreased extraocular motility. An MRI in December 2024 showed grossly stable bilateral enhancing orbital masses and persistent associated ocular proptosis but with prominence in both medial and lateral rectus muscles of bilateral orbits, suggesting multifocal involvement. Viable options were discussed with the patient, including debulking surgery with an oculoplastic surgeon or proton radiation therapy, but the patient elected to continue observation.

## 3 Discussion

Perineuroma of the bilateral orbits is a rare location to an already uncommon benign tumor. There have been few case reports covering PNSTs arising in the orbit with even fewer specifically on perineuromas of the orbit.

One such study presented the case of a 7-week-old boy who was found to have a sclerosing perineuroma of the orbit and symptoms of dacryocystitis (2). The presenting symptom in this case was a mass in the medial canthus of the left eye. This was a unique presentation in part because the extraneural subtype is more common in adults, whereas intraneural perineuroma is more common in adolescents (1). A similar lesion was found in a 17-year-old man who presented with a mass in the lateral aspect of the left upper eyelid, which was later found to be a soft tissue perineuroma of the lacrimal gland (3). Interestingly, recurrent dacryocystitis was also the presenting symptom for an 83-year-old woman with an orbital perineuroma that stained positive for Claudin-1, GLUT-1, and EMA (4). Another reported case was a 45-year-old man with increasing proptosis over 1 year, who was found to have intraconal mass on CT. Biopsy immunohistochemistry was negative for S-100 and weakly positive for EMA, confirming a diagnosis of soft tissue perineuroma. The above cases are all unilateral presentations of perineuroma.

There are currently no reports of bilateral perineuroma of the orbit; however, there have been cases of bilateral neurofibromas of the orbit (5). Additionally, there are reports of hybrid peripheral nerve sheath tumors (HPNSTs) occurring in the orbit. For example, one such case involved a 52-year-old woman who had right proptosis and pain and who was found to have a schwannoma–perineuroma HPNST (6). Another similar case evaluated a 78-year-old man presenting with left exophthalmos and who was later found to have a neurofibroma–schwannoma HPNST orbital mass (7).

Our case report on bilateral perineuromas demonstrated an extraneural soft tissue perineuroma (ESTP), one of the two perineuroma subtypes, which demonstrate characteristic spindle cells (1). Additionally, immunostaining is often positive for EMA, collagen type IV, laminin, and vimentin, but negative for S100 (1). This fits with the patient’s histology, which was positive for EMA and negative for S100. Studies have reported CD34 positivity in 64% of perineuroma samples (8), which was the other marker found positive for this patient.

It has been reported that extraneural perineuromas may be associated with specific NF1 and NF2 mutations, and intraneural perineuromas frequently contain TRAF7 mutations (9). However, none of the previous case reports examined, including this case, had the genetic component identified.

Generally, perineuromas are benign and curative via surgical resection (1). Owing to the bilateral orbital involvement for this patient, treatment remains difficult. Surgery or radiation in this area poses the risk of vision loss. A steroid taper only provided minimal and temporary relief of the weakness. For this patient with worsening symptoms, orbital decompression surgery is being considered to relieve the pressure caused by the mass.

To our knowledge, this is the first reported case of bilateral perineuroma of the orbits. The management of these bilateral and benign yet symptomatic orbital lesions presents a challenge. As there was minimal response to systemic treatment, this leaves surgery or radiation management as primary treatment options, both of which pose the risk of visual loss.

## Data Availability

The original contributions presented in the study are included in the article/**Supplementary Material**. Further inquiries can be directed to the corresponding author.

## Supplementary material

Supplementary Figure 1 Visual field photos for the right eye **(A)** and left eye **(B)**.
