# Supplementary figures and images for: Case Report: Bilateral perineuroma of the orbit presenting as vertical binocular diplopia
Source: Front Ophthalmol (Lausanne). 2026 Mar 13;6:1777572. doi: 10.3389/fopht.2026.1777572 (PMC13021456; doi:10.3389/fopht.2026.1777572)

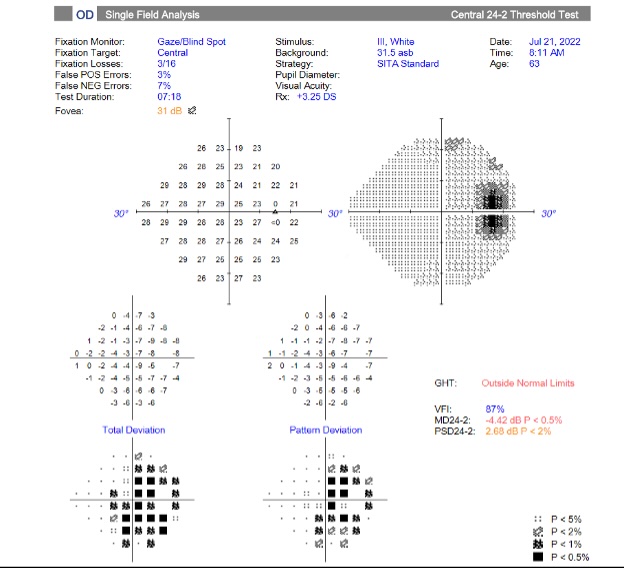

Supplement: Supplementary Figure 1 — Visual field photos for the right eye (A) and left eye (B). [file Image1.jpeg]

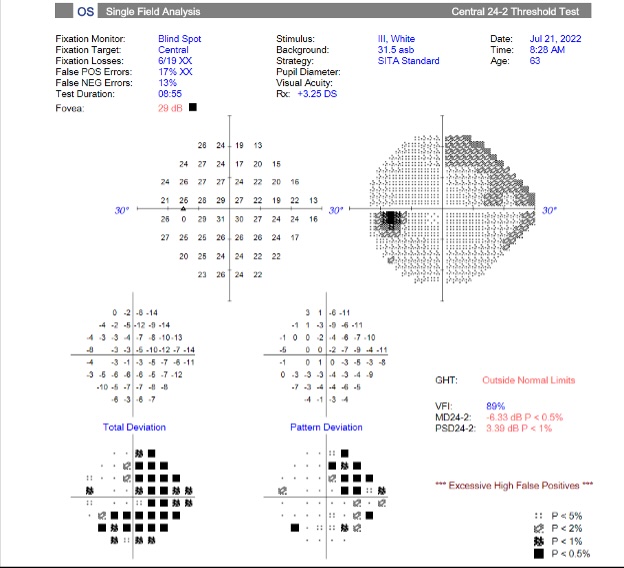

Supplement: Supplementary file 2 [file Image2.jpeg]
